# Personality Characteristics of Mothers of Children with Attention Deficit Hyperactivity Disorder as Assessed by the Minnesota Multiphasic Personality Inventory

**DOI:** 10.4306/pi.2008.5.4.228

**Published:** 2008-12-31

**Authors:** Soon Jeong Lee, Jung-Hwa Kwon, Yu Jin Lee

**Affiliations:** 1 Department of Psychiatry, Seoul Metropolitan Eunpyong Hospital, Seoul, Korea.; 2 Department of Psychiatry, Gachon University Gil Hospital, Incheon, Korea.

**Keywords:** Attention deficit hyperactivity disorder, Mother, Personality, Minnesota Multiphasic Personality Inventory

## Abstract

**Objective:**

The current study investigated the personality characteristics of mothers of children with attention deficit hyperactivity disorder (ADHD) using the Minnesota Multiphasic Personality Inventory (MMPI).

**Methods:**

Fifty mothers (average age of 38.1±4.2 years) of children with ADHD not having comorbidity (37 boys, 13 girls; average age of 8.5±1.9 years) and 59 mothers (average age of 38.1±2.7 years) of comparison children (37 boys, 13 girls; average age of 8.1±1.5 years) completed the Korean version of the MMPI. Only mothers whose psychiatric health was verified by the Structured Clinical Interview for axis-I DSM-IV disorders (SCID-IV) were included in current study.

**Results:**

After controlling for maternal age, maternal education level, children's gender, age, and total and verbal intelligence quotient (IQ), the MMPI scores of the mothers of children with ADHD were significantly higher on the depression (D), hysteria (Hy) and psychasthenia (Pt) scales than those of the mothers of children in the comparison group.

**Conclusion:**

These results suggested that even psychologically healthy mothers of children with ADHD alone might be depressed, histrionic and anxious.

## Introduction

A number of previous studies have noted an increased incidence of psychopathology and personality disorder in parents of children with attention deficit hyperactivity disorder (ADHD).^1^-^3^ However, they demonstrated that parents of children with ADHD comorbid with conduct disorder and oppositional defiant disorder were more likely to have mood disorders, anxiety disorders, antisocial personalities and engage in substance abuse.^1^ In another study, conduct disorder but not ADHD was reported to be linked to parental psychopathology by a structured psychiatric interview.^4^ Several previous studies used the Minnesota Multiphasic Personality Inventory (MMPI)^5^ to investigate the personalities of parents of children with ADHD, but their findings were inconsistent.^6^-^8^ Mothers of children with ADHD were previously reported to score the highest on the depression (D), hysteria (Hy) and psychopathic deviate (Pd) scales of the MMPI, but a comparison group was not included.^6^ Lahey (1989) noted that mothers of children with ADHD without conduct disorder did not score higher on any scale of the MMPI than the mothers of children in the comparison group, but suggested that coexisting conduct disorder could predict maternal personality disturbances.^7^ In anot her study, parents of children with ADHD showed higher Pd scores on their MMPI profiles than those in the community comparison group.^8^ These inconsistencies might result from the high prevalence of psychiatric illnesses among parents of children with ADHD and the lack of homogenous group of ADHD patients. To date, no study has measured the MMPI profiles of psychologically healthy mothers of children with ADHD not comorbid with conduct disorder or oppositional defiant disorder. In the current study, we aimed to examine the personality characteristics of mothers of children with ADHD using the MMPI.

## Methods

Fifty-six biological mothers of outpatients in the Seoul Metropolitan Eunpyeong Hospital with Diagnostic and Statistical Manual-fourth edition (DSM-IV)^9^-diagnosed ADHD and 63 biological mothers of comparison children who responded to advertisements for this study were recruited. Children with ADHD comorbid with other psychiatric disorders, including conduct disorder and oppositional defiant disorder, by semi-structured interviews and medical record reviews were not included in the present study. Comparison children had no psychiatric history, no current psychiatric symptoms and no current behavioral issues based on a semi-structured interview with DSM-IV criteria. The Structured Clinical Interview for axis-I DSM-IV disorders (SCID-IV)^10^ was administered to all mothers in order to evaluate their mental health. Due to the presence of current psychiatric disorders by SCID-IV or previous psychiatric history, six of the mothers in the ADHD group (dysthymia: 1, panic disorder: 1, major depressive disorder: 2, ADHD history: 1, and conduct disorder history: 1) and 4 of the mothers in the comparison group (dysthymia: 1, panic disorder: 1, generalized anxiety disorder: 1, and alcohol abuse history: 1) were excluded. Children with intelligence quotient (IQ) scores under 75 measured by the Korean Educational Development Institute-Wechsler Intelligence Scale for Children (KEDI-WISC) were excluded. None of the children had IQs below 80.

Finally, fifty mothers (average age of 38.1±4.2 years) of children with ADHD (37 boys, 13 girls; average age of 8.5±1.9 years) and 59 mothers (average age of 38.1±2.7 years) of comparison children (37 boys, 13 girls; average age of 8.1±1.5 years) completed the Korean version of the MMPI. The MMPI is one of the most frequently used tests for personality assessment. It was developed in 1943 as a screening tool for psychopathology and assessment of personality.^5^ It consists of 566 self-report true/false questions, which are scored for 8 clinical scales of psychopathology, 2 scales of personality dimensions i.e. masculinity-femininity (Mf) and social introversion (Si), and 3 validity scales. SPSS version 12.0 software was used for the statistical analysis.

## Results

The mean scores on all of the MMPI scales were within the normal limits for both groups of participants. There were no significant differences in maternal age, maternal educational level, or children's age between the ADHD and comparison groups (Independent t-test; p=0.933, p=0.773 and p=0.201 respectively, Table 1). There was no significant difference in the gender of the children between the two groups (Chi-square test; p=0.224, Table 1). The total and verbal IQ scores of the children with ADHD were significantly lower than those of the children in the comparison group (Independent t-test; p=0.023 and p=0.003 respectively; Table 1). After controlling for maternal age, maternal education, children's age, gender, and total and verbal IQ, the mothers of children with ADHD scored higher on the D, Hy and Pt scales (p=0.027, p=0.007 and p=0.037 respectively, Table 2).

## Discussion

To the best of our knowledge, this is the first study to assess the personality characteristics of mothers whose psychiatric health was verified using the SCID-IV. We also did not enroll children with ADHD who had other psychiatric comorbidities in the current study based on previous findings suggesting that there may be a strong association between coexisting conduct disorder and parental personality disorder.^7^

In the current study, the mothers of children with ADHD scored higher on the following MMPI scales: D, which measures depression; Hy, which measures hysterical reactions and denial of stressful situations; and Pt, which measures anxiety, than those of the comparison children. These findings support those of previous studies reporting that mothers of children with ADHD tended to be more depressed, self-blaming and socially isolated.^11^ They are also consistent with a previous finding demonstrating an increased risk of mood and anxiety disorders in parents of children with ADHD.^12^ However, Lahey et al. did not find higher MMPI scale scores in mothers of children with ADHD, as compared to those in the control group.^7^ We found strong statistical significance for the higher Hy scores on the MMPI profiles of mothers of children with ADHD. However, a prior study, which assessed psychopathology using a structured interview, suggested a relationship between maternal hysteria and conduct disorder, but not ADHD.^4^ An objective personality test, such as the MMPI, might be more sensitive to subclinical personality traits than the formal diagnosis of a disorder by clinical interview.^7^ These discrepancies could be due to the strength of the current study, which had a larger sample size, was exclusive to mothers with psychiatric disorders, and controlled for more possible confounding factors in the statistical analysis.

In the current study, the mothers of the children with ADHD did not score significantly high on the Pd scale of the MMPI. A higher Pd score was a consistent finding in two previous family studies that measured the parental MMPIs of children with ADHD.^6^,^8^ A plausible explanation for this discrepancy might be the exclusion of children with ADHD comorbid with conduct disorder and oppositional defiant disorder in the current study. The notion that an antisocial personality in men and hysteria in women may share the same origin,^13^ rather than elevated Pd score, would be another plausible explanation for the higher Hy scores in the current study, which included only female subjects.

Many aspects of personality are known to be under partial genetic influence. In a prior study, the heritability estimates of MMPI scores were from 0.26 to 0.61.^14^ Therefore, the higher D and Pt scores on the maternal MMPI profiles in the current study might support previous notion that children with ADHD were highly likely to have comorbid anxiety and depression and that they have more difficulties in their social relationships.^15^ In addition, ADHD itself has been suggested to have a large genetic component.^16^

However, a non-genetic understanding of these findings is possible. The MMPI scores could be more state-dependent than trait-dependent in the individual's lifetime.^8^ Our findings of more depression, hysteria and anxiety on the MMPI could reflect the maternal psychological burden of raising a child with a chronic behavioral disorder rather than a suggestion of personality characteristics with a genetic predisposition to ADHD.

Parents play an important role in delivering the proper treatment to children with ADHD. It would not be surprising that maternal depression is related to their poor parenting behavior.^17^ Parental depression, marital distress and parental skills were noted to have a negative effect on treatment compliance in children with ADHD.^18^ Therefore, the evaluation and proper psychiatric interventions for mothers of children with ADHD could be crucial factors in the treatment process, and an understanding of maternal personality characteristics might be helpful for improving the prognosis of children with ADHD.

The present study has several limitations, including its relatively small sample size and the lack of a parametric assessment investigating the severity of psychopathology in the mothers or children. As conduct behaviors in ADHD could become an issue in later in life, the exclusion of children with ADHD and behavioral problems might not be perfect. In addition, the total and verbal IQ scores of children with ADHD were significantly lower than those of the comparison children. This finding could support the previous findings that the mean IQ scores of children with ADHD were lower than those of normal controls and that the IQ scores correlated negatively with ADHD symptoms.^19^,^20^ To rule out the potential effect of the child's intelligence on the MMPI profile of the mother, we controlled for total and verbal IQ score as a confounding factor in the statistical analysis.

In conclusion, these results suggested that even psychologically healthy mothers of children with ADHD alone might have a characteristic personality, which was observed to be more depressed, more histrionic and more anxious.

## Figures and Tables

**TABLE 1:**
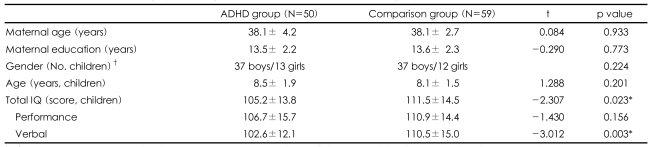
Comparison of demographic findings between the ADHD and comparison groups

Independent t-test, ^*^p<0.05, ^†^Chi-square test. ADHD: attention deficit hyperactivity disorder, IQ: intelligence quotient

**TABLE 2:**
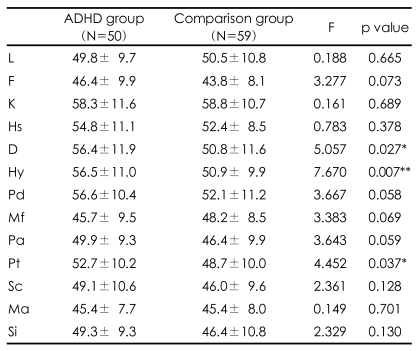
Comparison of the MMPI scores of mothers of children in the ADHD and comparison groups

ANCOVA controlling for maternal age, children's age, gender, total IQ and verbal IQ by general linear model in SPSS 12.0, ^*^p<0.05, ^**^p<0.01. MMPI: Minnesota Multiphasic Personality Inventory, ADHD: attention deficit hyperactivity disorder, L: lie, F: infrequency, K: defensiveness, Hs: hypochondriasis, D: depression, Hy: hysteria, Pd: psychopathic deviate, Mf: masculinity-femininity, Pa: paranoia, Pt: psychasthenia, Sc: schizophrenia, Ma: hypomania, Si: social introversion
